# A Unified Complex-Fresnel Model for Physically Based Long-Wave Infrared Imaging and Simulation

**DOI:** 10.3390/jimaging12010033

**Published:** 2026-01-07

**Authors:** Peter ter Heerdt, William Keustermans, Ivan De Boi, Steve Vanlanduit

**Affiliations:** InViLab Research Group, University of Antwerp, Groenenborgerlaan 171, 2020 Antwerpen, Belgium; peter.terheerdt@uantwerpen.be (P.t.H.); william.keustermans@uantwerpen.be (W.K.); ivan.deboi@uantwerpen.be (I.D.B.)

**Keywords:** spectral rendering, infrared imaging, complex refractive indices

## Abstract

Accurate modelling of reflection, transmission, absorption, and emission at material interfaces is essential for infrared imaging, rendering, and the simulation of optical and sensing systems. This need is particularly pronounced across the short-wave to long-wave infrared (SWIR–LWIR) spectrum, where many materials exhibit dispersion- and wavelength-dependent attenuation described by complex refractive indices. In this work, we introduce a unified formulation of the full Fresnel equations that directly incorporates wavelength-dependent complex refractive-index data and provides physically consistent interface behaviour for both dielectrics and conductors. The approach reformulates the classical Fresnel expressions to eliminate sign ambiguities and numerical instabilities, resulting in a stable evaluation across incidence angles and for strongly absorbing materials. We demonstrate the model through spectral-rendering simulations that illustrate realistic reflectance and transmittance behaviour for materials with different infrared optical properties. To assess its suitability for thermal-infrared applications, we also compare the simulated long-wave emission of a heated glass sphere with measurements from a LWIR camera. The agreement between measured and simulated radiometric trends indicates that the proposed formulation offers a practical and physically grounded tool for wavelength-parametric interface modelling in infrared imaging, supporting applications in spectral rendering, synthetic data generation, and infrared system analysis.

## 1. Introduction

Infrared imaging and thermography are widely used for non-contact inspection, condition monitoring, and machine vision in domains such as structural health assessment, composites, electrical systems, aerospace, and energy. Reviews have shown how infrared thermography (IRT) has evolved into a mature non-destructive evaluation (NDE) technique central to condition-based maintenance [1,2]. Recent integration of IRT with computer vision and deep learning has enabled powerful data-driven defect detection systems while increasing the need for large, physically meaningful datasets [3,4]. At the same time, active thermography methods—such as pulsed phase and lock-in thermography—have become quantitative tools [2,5] and are now often combined with computer vision and numerical simulation and inverse modelling for defect sizing, material characterization, and model-assisted AI [6,7]. Beyond classical thermography, the infrared community has increasingly focused on synthetic infrared image generation for sensor design, algorithm benchmarking, and remote sensing. Large-scale simulators such as DIRSIG and LESS produce multi- and hyperspectral, polarimetric, and thermal infrared imagery of complex scenes for system-level analysis [8,9]. A recent survey highlights these physics-based simulators—alongside deep learning-based methods—as essential tools for training and validating surveillance, remote-sensing, and target-recognition systems, especially when real data are expensive or difficult to obtain [10].

At the same time, Blender has become a popular open-source platform for synthetic dataset generation, including in the thermal infrared. Several approaches construct 3D geometry in Blender and map thermal textures generated by GANs or other image-to-image translation models trained on paired visible/thermal data [11]. Other pipelines combine real thermal backgrounds with synthetic characters rendered using simplified radiometric shaders, or use Blender-based workflows to augment UAV thermal datasets for search-and-rescue and aerial monitoring [12,13]. While effective, these methods typically employ heuristic emissivities, temperature textures or single-band greyscale representations, and do not exploit wavelength-dependent complex refractive indices to model reflection, transmission, and absorption in a physically consistent manner. In addition to wavelength-dependent optical constants, infrared simulation must also account for the temperature of objects, since thermal emission depends on both emissivity and the Planck distribution. While the complex refractive index determines how a surface reflects and absorbs incident light, temperature governs the magnitude of its self-emitted radiance. Accurate LWIR modelling therefore requires combining Fresnel-based emissivity with temperature-dependent thermal radiation.

Meanwhile, high-quality spectral optical constants n(λ) and k(λ) have become increasingly available for a wide variety of infrared-relevant materials, either from curated databases or from inverse-model-based retrieval algorithms applied to reflectance/transmittance measurements [14,15]. This availability creates an opportunity: if complex refractive indices could be used directly within a standard 3D rendering environment, one could construct virtual infrared “digital twins” of experimental setups using only geometry and wavelength-dependent optical constants, and simulate realistic spectral images for arbitrary infrared camera configurations.

To address this gap, we introduce a novel Blender node for spectral rendering that models reflection, transmission, and absorption across the electromagnetic spectrum using wavelength-dependent complex refractive indices as the sole material input. For simulations at thermal wavelengths we also model the emission from the calculated reflectance and transmission (assuming that the temperature is known). This Blender-integrated approach offers several advantages over existing thermography-oriented simulation workflows and specialized radiative-transfer codes. First, it leverages Blender’s mature ecosystem for geometry modelling, animation and camera control, facilitating adoption by thermography practitioners, robotics researchers and machine vision users already relying on Blender for synthetic data generation. Second, by relying exclusively on complex refractive indices, it provides a consistent interface between measured optical constants and simulation, and naturally supports reflective/transmissive materials, multilayer coatings and dispersive behaviour across the NIR–SWIR–MWIR–LWIR range. Third, a single scene description can support thermography experiment design, multispectral camera modelling, synthetic data generation for deep learning, and lightweight remote-sensing scene simulation.

Because realistic infrared rendering depends on correctly modelling reflection, transmission, and absorption at material interfaces, our Blender node adopts a full Fresnel formulation, ensuring that these interactions are treated consistently across all wavelengths. Most existing Fresnel implementations in rendering and IR simulation rely on RGB-based approximations or direct textbook formulas that do not robustly handle complex refractive indices. When extended to infrared materials—where absorption is strong and *k* can be large—these simplified models often exhibit sign ambiguities, branch-cut inconsistencies, and numerical instabilities, possibly leading to non-physical results. We introduce a unified, wavelength-driven Fresnel model that avoids these issues by using complex refractive indices directly, enabling stable and physically consistent reflection, transmission, and absorption across the full spectrum and providing a flexible framework for infrared simulation and rendering within Blender.

The remainder of this paper is organized as follows. Section 2 reviews the radiometric and optical models underlying the approach and gives an overview of related work. Section 3 presents the outline of our unified model, and Section 4 describes the Blender implementation. Section 5 presents case studies of comparisons of the simulation results with databases and experimental data. Section 6 discusses limitations and possible extensions and draws concludes.

## 2. Background and Related Work

### 2.1. Related Work

Many existing Fresnel implementations in computer graphics and infrared simulation rely on simplified or numerically fragile formulations that become unreliable when extended to wavelength-dependent complex refractive indices in the infrared. Microfacet BRDF/BTDF models and GPU-oriented renderers typically use Schlick-type RGB approximations or directly apply textbook Fresnel equations without addressing the subtleties of complex arithmetic, such as correct branch selection for square roots and logarithms [16,17]. In the IR domain—where absorption is strong and k(λ) may be large—these shortcuts lead to well-known issues, including sign ambiguities in the longitudinal wave-vector and in the retrieval of *n* and *k* [18], numerical instabilities near grazing incidence or in highly absorbing media, and inconsistencies in branch-cut choices for multilayer stacks. Although recent work in spectral and display-oriented rendering has begun to expose how Fresnel computations are implemented in modern pipelines, these systems remain optimized for the visible spectrum and wide-gamut RGB workflows, and rarely address IR-specific numerical pathologies or guarantee energy-conserving, causal behavior across the full complex frequency plane [19,20].

Spectral rendering has attracted renewed interest—particularly in combination with wave-optics methods [21,22]—but most existing approaches are highly specialized and operate over limited spectral ranges. Foundational work [23] and recent surveys [24] emphasize the importance of physically-based spectral simulation for accurate material appearance and optical research, building on classical formulations of radiometry and interface physics [25,26]. However, despite the widespread adoption of production-grade 3D engines such as Unity3D, Unreal Engine and Blender in vision, robotics and simulation research [27,28,29], their material systems remain predominantly RGB-focused and offer limited support for physically correct Fresnel behavior outside the visible range.

To our knowledge, no previous work provides a unified, wavelength-driven Fresnel model that operates coherently across the full electromagnetic spectrum within a mainstream 3D rendering environment. The approach presented in this paper fills this gap: we introduce a physically complete yet computationally efficient spectral Fresnel node that directly consumes wavelength-dependent complex refractive indices, consistently handles conductors and dielectrics, and avoids the numerical ambiguities present in existing implementations. This enables accurate reflection, transmission, and absorption modelling from the UV to the LWIR within Blender’s rendering pipeline, offering capabilities not previously available in general-purpose 3D software. It is important to emphasize that the physical principles underlying complex Fresnel reflection and transmission are well established in classical electromagnetics and optics. The contribution of this work does not lie in introducing new Fresnel theory, but in providing a numerically robust and practically usable reformulation that enables wavelength-dependent complex refractive indices to be employed reliably within a mainstream 3D rendering environment. By explicitly enforcing the physically admissible Fresnel branch and avoiding common numerical ambiguities, the proposed formulation bridges the gap between established electromagnetic theory and practical infrared rendering and simulation workflows.

### 2.2. Fresnel Theory for Infrared Materials

At the heart of our approach lies the use of full, complex Fresnel reflection and refraction equations, which are fundamental for accurately modelling light interactions at the interface between two media. These equations are derived from the principles of electromagnetism and use complex IORs, which are wavelength-dependent. By leveraging the full, complex calculations of Fresnel reflection and refraction, we can handle a wide range of materials, including both lossy and lossless media, in a physically correct manner. This approach is crucial for unifying material models that would typically be treated separately, such as glass and metallic materials, into a single, generalized material shader.

We draw from prior work on harmonic inhomogeneous plane waves (HIPWs), Refs. [30,31,32,33,34] that provides a more general framework for Fresnel calculations at the interface between two lossy media. These works extend traditional models by allowing for a broader class of material interactions, particularly in complex and inhomogeneous environments. However, our method remains within the traditional computer graphics context, where we continue to treat light as homogeneous plane waves, a simplification that is computationally efficient and widely used in graphics rendering.

Material nodes describing dielectric materials, like glass, water or diamond, are different from the nodes describing metallic materials, like for instance gold, copper or aluminum. Additionally, most physically based production renderers adopt the widely used Schlick approximation for the calculation of the Fresnel reflectance and transmittance in dielectrics and certainly use approximations for metallic materials [35]. By using a clear full Fresnel formulation, we can now represent both glass-like and metallic materials using a single “Full Fresnel” shader. This unification of materials is particularly important in the context of spectral rendering, where traditional shaders for different material types must be separately defined for each spectral domain (e.g., VIS, NIR, SWIR). Our approach eliminates this complexity by allowing the same shader to handle all these domains, ensuring a consistent and seamless transition between them by simply adjusting the wavelength-dependent parameters.

### 2.3. Fresnel Reflectance and Transmittance

In optics the famous Fresnel equations for reflection and refraction describe the amount of energy that gets reflected and refracted at the specular boundary of two interfaces with different indices of refraction. Derivation of these equations, starting with the formulation of Maxwell’s equations in linear, homogeneous, isotropic and non-dispersive media, can be found in many classic textbooks on modern optics [25,26,36]. At the interface of two lossless (non-absorbing) dielectrics the following equations are found:

(1) $$\begin{array}{cc} \; & r_{⊥}=\frac{\cos\theta_{i}-\sqrt{n^{2}-\sin^{2}\theta_{i}}}{\cos\theta_{i}+\sqrt{n^{2}-\sin^{2}\theta_{i}}}\\ \; & r_{\|}=\frac{n^{2}\cos\theta_{i}-\sqrt{n^{2}-\sin^{2}\theta_{i}}}{n^{2}\cos\theta_{i}+\sqrt{n^{2}-\sin^{2}\theta_{i}}}\\ \; & t_{⊥}=\frac{2\cos\theta_{i}}{\cos\theta_{i}+\sqrt{n^{2}-\sin^{2}\theta_{i}}}\\ \; & t_{\|}=\frac{2n\cos\theta_{i}}{n^{2}\cos\theta_{i}+\sqrt{n^{2}-\sin^{2}\theta_{i}}} \end{array}$$

**Figure 1:**
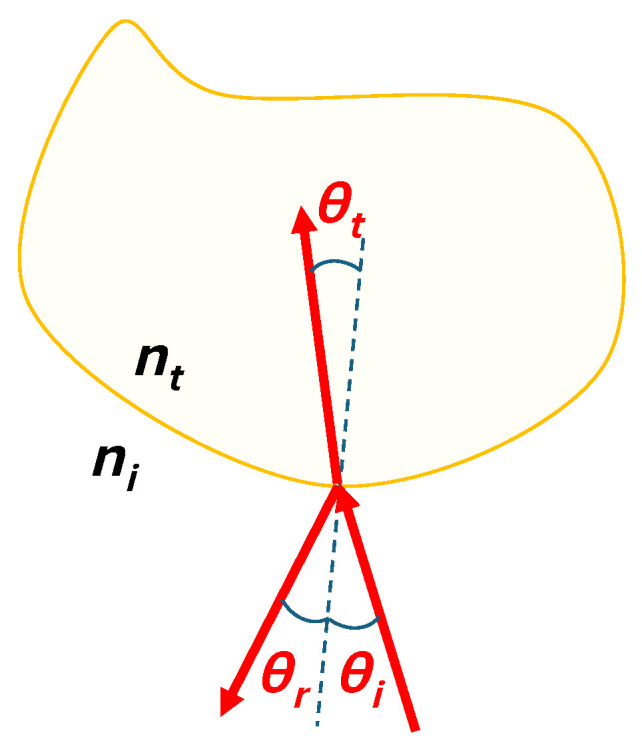
Classic Fresnel reflection and refraction at the boundary of two media with indices of refraction $n_{i}$ and $n_{t}$. The dashed line represents the normal to the boundary.

Here $r_{⊥}$ and $r_{\|}$ are the complex amplitude reflectances for the transverse electric (TE) and transverse magnetic (TM) polarizations respectively and completely similar $t_{⊥}$ and $t_{\|}$ represent the complex amplitude transmittances for the TE and TM polarizations. The term $n\left(\lambda \right)=\frac{n_{t}\left(\lambda \right)}{n_{i}\left(\lambda \right)}$ is the ratio between the refractive index of the medium the light transmits to (hence the subscript *t*), and the refractive index of the medium where the light is incoming towards the interface (hence the subscript *i*). Here λ expresses the wavelength dependence of n. Finally $\theta_{i}$ is the incoming angle of the light with respect to the local surface normal. This is visualized in Figure 1.

However, when the environments, where the light travels through, show absorption, a different scenario arises. In general these media are represented with a complex index of refraction, yet still wavelength dependent, where the imaginary part accounts for the amount of absorption of the medium. Metals like gold, copper or aluminum form a clear example of highly absorbing media. If both the incoming and transmitted medium are lossy their respective IORs will be:

(2) $$\begin{array}{c} \tilde{n_{i}}\left(\lambda \right)=n_{i}\left(\lambda \right)+k_{i}\left(\lambda \right)j\\ \tilde{n_{t}}\left(\lambda \right)=n_{t}\left(\lambda \right)+k_{t}\left(\lambda \right)j \end{array}$$

The values of $k_{i}$ and $k_{t}$, the imaginary parts of the IORs, are a direct measure for the absorption in a medium. But a complex IOR means that the wave vector itself can actually become complex. A wave with such a complex wave vector is called a harmonic inhomogeneous plane wave (HIPW) and its use in solving the Maxwell equations for linear, homogeneous, isotropic and non-dispersive media leads to a more general formulation of Snell’s laws of reflection and refraction [30]. In turn this leads to the departure from the traditional equations for the Fresnel coefficients as presented in Formula (1). At the same time this general formulation also predicts that the wave fronts of constant phase do not necessarily travel in the same direction as the wave fronts of constant amplitude. Formulated differently this means that refraction and absorption happen in different directions for HIPW’s and even the plane of incidence doesn’t necessarily coincides with the plane of refraction. All these observations have stimulated an almost ongoing, interesting but confusing discussion on what the actual direction of refraction should be [30,31,32,33,34].

To proceed, the assumption is made that the plane waves remain homogeneous, meaning the planes of constant phase coincide with the planes of constant amplitude. The generalized Snell-Descartes laws then reduce to the ones describes in Equation (1), with the exception that we now introduce the complex IORs in the formulas:

(3) $$\begin{array}{cc} \; & r_{⊥}=\frac{\cos\theta_{i}-\sqrt{{\tilde{n}}^{2}-\sin^{2}\theta_{i}}}{\cos\theta_{i}+\sqrt{{\tilde{n}}^{2}-\sin^{2}\theta_{i}}}\\ \; & r_{\|}=\frac{\sqrt{{\tilde{n}}^{2}-\sin^{2}\theta_{i}}-{\tilde{n}}^{2}\cos\theta_{i}}{\sqrt{{\tilde{n}}^{2}-\sin^{2}\theta_{i}}+{\tilde{n}}^{2}\cos\theta_{i}}\\ \; & t_{⊥}=\frac{2\cos\theta_{i}}{\cos\theta_{i}+\sqrt{{\tilde{n}}^{2}-\sin^{2}\theta_{i}}}\\ \; & t_{\|}=\frac{2\tilde{n}\cos\theta_{i}}{{\tilde{n}}^{2}\cos\theta_{i}+\sqrt{{\tilde{n}}^{2}-\sin^{2}\theta_{i}}} \end{array}$$

From the complex amplitude reflectances $r_{⊥}$ and $r_{\|}$ and the transverse polarizations $t_{⊥}$ and $t_{\|}$, the perpendicular and parallel reflectance can be calculated:

(4) $$R_{⊥}=r_{⊥}r_{⊥}^{*},\;R_{\|}=r_{\|}r_{\|}^{*},$$

### 2.4. Thermal Emission and Temperature Dependence

While the Fresnel formulation determines how incident radiation is reflected, transmitted, and absorbed at an interface, infrared rendering also requires modelling the material’s own thermal emission. In contrast to purely reflective optical simulations, thermal radiation depends not only on the complex refractive index $\tilde{n}\left(\lambda \right)=n\left(\lambda \right)+ik\left(\lambda \right)$ but also on the local temperature *T*. The emitted spectral radiance is governed by Planck’s law,

(5) $$L_{\mathrm{emit}}\left(\lambda ,T\right)=\varepsilon \left(\lambda ,\theta \right)\;L_{\mathrm{bb}}\left(\lambda ,T\right),$$

where $L_{\mathrm{bb}}\left(\lambda ,T\right)$ is the blackbody spectral radiance and ε(λ,θ) is the directional emissivity. Since emissivity is linked to the Fresnel reflectance through Kirchhoff’s law,

(6) ε(λ,θ)=1−R(λ,θ),

the temperature *T* enters the simulation explicitly through the blackbody radiation term and implicitly through the angle- and wavelength-dependent Fresnel coefficients. Consequently, realistic LWIR simulations must combine (i) complex refractive indices for interface interactions and (ii) accurate temperature fields for thermal emission. This dual dependence is essential for reproducing the radiometric behaviour of heated objects and for generating physically meaningful synthetic LWIR images.

## 3. Unified Wavelength-Dependent Fresnel Formulation

The formulation presented in this section should be understood as a numerically stable reparameterization of the classical Fresnel equations for complex refractive indices, rather than as a new physical model. While the underlying Fresnel relations follow directly from Maxwell’s equations, their direct numerical evaluation in the presence of wavelength-dependent complex indices is known to suffer from sign ambiguities and instabilities. The proposed reformulation addresses these issues at the implementation level, ensuring continuous, energy-consistent, and physically admissible behavior across infrared wavelengths and incidence angles.

### 3.1. Fresnel Reflectance and Transmittance Ambiguities

When extending the Fresnel equations to complex refractive indices, one encounters the well-known sign ambiguity in the evaluation of the transmitted longitudinal wavevector $\sqrt{{\tilde{n}}^{2}-\sin^{2}\theta_{i}}$. As discussed in classical electromagnetic treatments [25,26], both branches of the complex square root formally satisfy the Helmholtz equation, but only one represents the physically admissible solution. For a passive medium, Maxwell’s boundary conditions require the transmitted field to decay with increasing depth, which imposes the constraint Im(kz)>0 on the longitudinal component of the wavevector [18]. Selecting the opposite branch results in an exponentially growing field inside the material, violating passivity and leading to non-physical behaviour of the Fresnel coefficients. In practice, an incorrect branch choice may manifest as discontinuities of the reflectance across wavelength, numerical instabilities near grazing incidence, or even violations of energy conservation (e.g., reflectances exceeding unity). In multilayer configurations, such inconsistencies compound across interfaces and can produce artificial phase jumps or spurious spectral oscillations. For this reason, stable and physically consistent Fresnel calculations require explicit enforcement of the physically correct branch—typically through passivity or causality constraints—to guarantee continuous, energy-conserving, and numerically robust reflectance and transmittance values.

### 3.2. Proposed Fresnel Formulation

To resolve the sign ambiguity inherent in the complex square-root term of the Fresnel equations, our method reformulates $\sqrt{{\tilde{n}}^{2}-\sin^{2}\theta_{i}}$ using auxiliary quantities (*A* and *B*) in the derivation of the perpendicular and parallel reflectance such that only strictly non-negative components are included, ensuring that the physically admissible branch (corresponding to a decaying transmitted field in passive media) is selected automatically. As a result, the Fresnel coefficients remain continuous, numerically stable, and energy-consistent across all wavelengths and incidence angles. A brief description of the method is given in this section, while the full derivation is given in Appendix A.

Starting from the standard complex Fresnel amplitudes, one would normally evaluate the transmitted longitudinal wavevector through the term $\sqrt{{\tilde{n}}^{2}-\sin^{2}\theta_{i}}$, which admits two algebraic branches and introduces a sign ambiguity that leads to numerical instabilities. To avoid this, we rewrite the square-root expression in terms of the auxiliary quantities *A* and *B*,

(7) $$\sqrt{{\tilde{n}}^{2}-\sin^{2}\theta_{i}}=A+jB,$$

where *A* and *B* are derived directly from the real quantities $ℜ({\tilde{n}}^{2}-\sin^{2}\theta_{i})$ and $ℑ({\tilde{n}}^{2}-\sin^{2}\theta_{i})$ (see Appendix A). By construction, *A* and *B* satisfy

(8) $$A=\sqrt{\frac{\left|Z|+ℜ(Z\right)}{2}},\;B=\mathrm{sgn}\;\left(ℑ(Z)\right)\sqrt{\frac{\left|Z|-ℜ(Z\right)}{2}},\;Z={\tilde{n}}^{2}-\sin^{2}\theta_{i},$$

ensuring that the physically admissible branch (with $ℑ(k_{z})>0$ in passive media) is selected automatically. Substituting A+jB into the perpendicular and parallel Fresnel coefficients in Equation (3) yields compact expressions for $r_{⊥}$ and $r_{\|}$ without any ambiguous square roots. The reflectances then follow directly as

(9) $$R_{⊥}=r_{⊥}r_{⊥}^{*},\;R_{\|}=r_{\|}r_{\|}^{*},$$

providing stable and energy-consistent results across all wavelengths and incidence angles.

Although the underlying physics is classical, the practical significance of this reformulation lies in its numerical behavior: across all tested wavelengths, incidence angles, and absorption regimes, the Fresnel coefficients remain continuous and free of branch-related discontinuities that commonly arise in naive implementations.

For comparison, it is worth noting that a naive numerical evaluation of the complex Fresnel equations—obtained by directly computing the complex square root in $\sqrt{{\tilde{n}}^{2}-\sin^{2}\theta_{i}}$ without enforcing passivity—admits two algebraic branches. In practice, this often leads to discontinuities in the Fresnel coefficients, reflectance values exceeding unity, or abrupt sign changes near grazing incidence or in strongly absorbing infrared materials. By explicitly reformulating the square-root term in terms of the auxiliary quantities *A* and *B*, the present approach removes this ambiguity and guarantees continuous, energy-consistent behavior across all tested angles and wavelengths.

## 4. Implementation Framework

### 4.1. Rationale for Using Blender

Unity and Unreal Engine are widely used for real-time rendering, procedural scene generation, and sensor simulation. However, Blender offers a distinct advantage for scientific applications due to its fully open-source architecture. Beyond the conceptual benefit of transparency, this openness enables direct modification of the rendering pipeline and the development of custom extensions—capabilities not available in closed commercial engines.

Of particular relevance to infrared research, Blender’s Cycles renderer provides a modular shading system in which new material-interaction nodes can be inserted. This allows the implementation of physically based optical models that go beyond standard RGB workflows, enabling, for example, wavelength-dependent scattering, absorption, or refractive-index-driven behavior. Such flexibility makes Blender a valuable platform for simulating complex light–matter interactions, generating physics-grounded synthetic datasets, and integrating external spectral or thermophysical data into scene rendering. Consequently, Blender functions not only as a 3D modelling and animation environment but also as a customizable scientific visualization tool well suited for infrared imaging, thermography research, and multispectral system simulation.

### 4.2. Fresnel as Building Block

The motivation for revisiting the Fresnel calculations in this work lies in the fact that Fresnel behavior fundamentally determines how materials reflect and transmit light, and thus underpins most physically based shading models used in Blender and other production renderers. Instead of treating metals and dielectrics as separate shader classes, our unified formulation demonstrates that both optical responses emerge directly from the same physical principles. In practice, many renderers rely on Schlick-type approximations for dielectrics and conductors [35], while more recent work proposes refinements for metallic coatings [37]. Other approaches typically use approximations tailored to individual material classes, reinforcing the need for a more general and physically consistent treatment.

**Figure 2:**
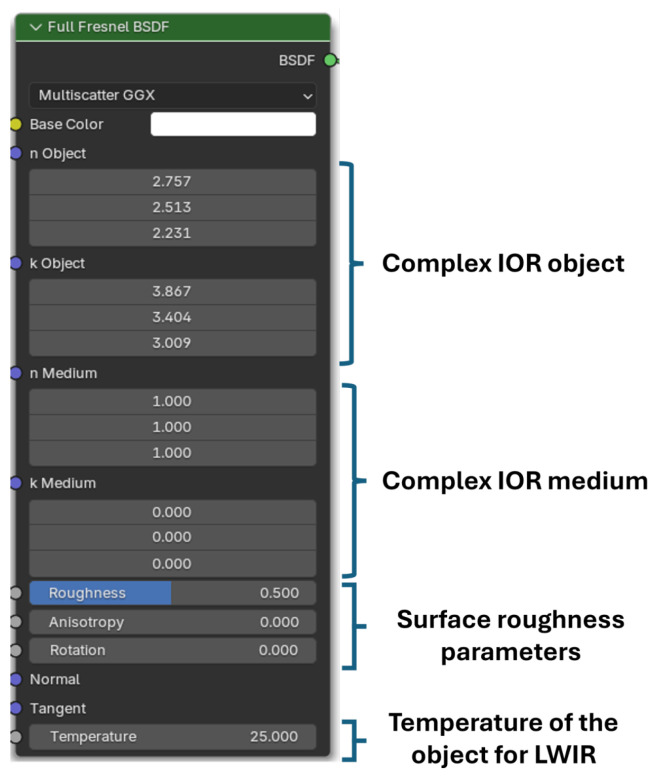
The full Fresnel surface shader material node, proposed in this work, as implemented in Blender 4.4 source code.

Figure 2 illustrates the custom Blender node used in this study, which encapsulates the full Fresnel surface interaction. The node accepts complex refractive indices for both the material and surrounding medium, along with a surface-roughness parameter, and supports three-channel inputs to model absorption and dispersion across the visible spectrum. All renderings were performed on a Lenovo ThinkBook 16 G6 IRL.

### 4.3. Blender Code

All implementation-level code integrated into the Blender source is beyond the scope of this article. However, the scripts used to generate the rendered results, along with all example .blend files, are available upon request. In this work, we modified the Blender 4.4 source code to introduce a new material surface shader—internally referred to as the Full Fresnel Shader—based on the unified Fresnel formulation described earlier. Although Blender 4.4 includes a recently introduced metallic shader that accepts real and complex refractive indices, its reflectance model uses a metal-specific approximation. Along with Blender’s existing Glass and Principled BSDF nodes, this provides a useful baseline for comparing the proposed unified Fresnel model against the conventional, material-specific approaches.

## 5. Simulation Examples and Experimental Validation

**Figure 3:**
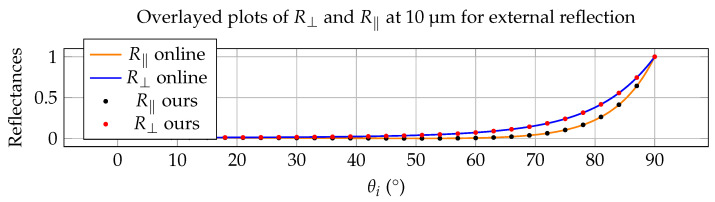
Comparison with online data for water at 10 µm in external reflection mode (air to water).

**Figure 4:**
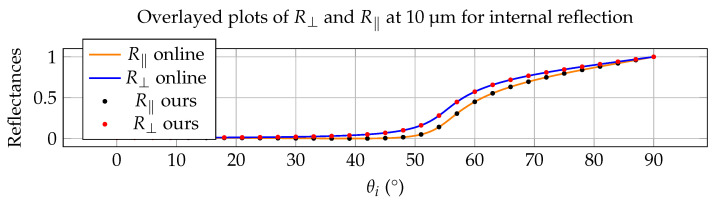
Comparison with online data for water at 10 µm in internal reflection mode (water to air).

**Figure 5:**
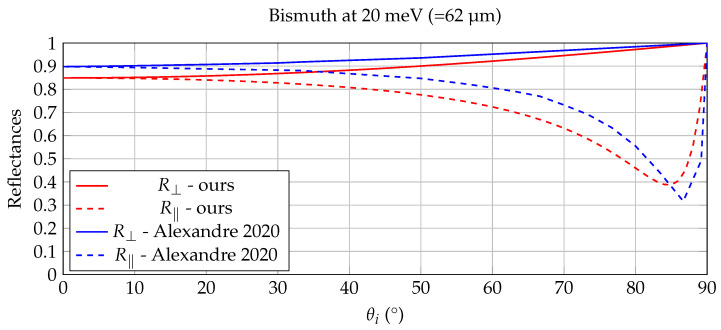
Comparison of the Bismuth reflectances from [40] with our full Fresnel model at 20 meV. The reflectances calculated with the full Fresnel model, after conversion of the dielectric values in [39] to ($n_{t}$, $k_{t}$)-values by Equation (13), closely resemble the model in [40].

**Figure 6:**
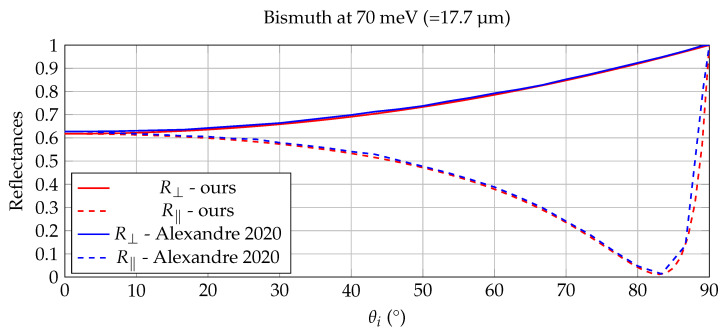
Comparison of the Bismuth reflectances from [40] with our full Fresnel model at 70 meV. The reflectances calculated with the full Fresnel model, after conversion of the dielectric values in [39] to ($n_{t}$, $k_{t}$)-values by Equation (13), closely resemble the model in [40].

Throughout the validation examples in this section, the full complex refractive index $\tilde{n}\left(\lambda \right)=n\left(\lambda \right)+ik\left(\lambda \right)$ is used in the Fresnel calculations. The imaginary component k(λ) is essential for accurately modeling absorption, angular reflectance, and emissivity in infrared materials. Although the experimental results are not presented explicitly in terms of complex refractive indices, their influence is implicit in the measured reflectance and thermal-radiometric behavior shown in Figure 3, Figure 4, Figure 5 and Figure 6.

### 5.1. Validation Against an Optical-Constant Database

Or method has been validated against several materials in online databases. Here with include as an example the case of the interaction between air and water with materials from the database described in Polyanskiy et al. [38] (see https://refractiveindex.info/ (accessed on 7 November 2025). We consider $R_{⊥}$ and $R_{\|}$ for water at a wavelength of 10 µm (LWIR) and a complex IOR of water equal to $n_{H_{2}O}$ = 1.218 and $k_{H_{2}O}$ = 0.0508. Our approach is compared to online calculations and again this resulted in a perfect match, both for external as well as internal reflections. This is depicted in Figure 3 and Figure 4. One important thing to notice is, that because of the significantly higher $k_{H_{2}O}$-value, the internal reflection curve in Figure 4 doesn’t show a steep transition to total reflection, where $R_{⊥}$, $R_{\|}$ and $R_{N}$ are always equal to 1. Instead for every incoming angle $\theta_{i}$ there is a corresponding reflectance value different from 1. This confirms the results in [31,32], which suggested the disappearance of total internal reflection in media where significant absorption is present.

### 5.2. Benchmarking with Experimentally Measured Bismuth Optics

A nice working case for the validation of our calculations of $R_{⊥}$ and $R_{\|}$ is described in [39,40], where the optically appealing element Bismuth undergoes both a thorough theoretical as experimental study. In [39] the complex relative dielectric function $\tilde{\epsilon }=\epsilon_{1}+\epsilon_{2}j$ is measured experimentally and a superposition of Drude and Lorentz-like terms is accurately fitted to the experimental data. Axexandre et al. [40] uses this data to verify the outcome of their Fresnel model, which deals with the refraction from a lossless dielectric to a lossy conductor.

The relative dielectric function can be related to the complex IOR, through the following equation:

(10) $$\tilde{n}=n+kj=\sqrt{\epsilon_{1}+\epsilon_{2}j}$$

By squaring both sides and equating the corresponding real and imaginary parts, one gets:

(11) $$\begin{array}{cc} \; & \epsilon_{1}=n^{2}-k^{2}\\ \; & \epsilon_{2}=2nk \end{array}$$

Substituting $k=\frac{\epsilon_{2}}{2n}$ into the equation for $\epsilon_{1}$, results in a quadratic equation in $n^{2}$:

(12) $$n^{4}-n^{2}\epsilon_{1}-\frac{\epsilon_{2}^{2}}{4}=0$$

The roots of $n^{2}$ are than given by:

$$n^{2}=\frac{\epsilon_{1}\pm \sqrt{\epsilon_{1}^{2}+\epsilon_{2}^{2}}}{2}$$

where only the plus sign gives a valid solution, as n needs to be real. So we can calculate n and k from the real and imaginary parts of the relative dielectric function via:

(13) $$\begin{array}{cc} \; & n=\frac{\sqrt{2}}{2}\sqrt{\epsilon_{1}+\sqrt{\epsilon_{1}^{2}+\epsilon_{2}^{2}}}\\ \; & k=\frac{\epsilon_{2}}{2n} \end{array}$$

**Figure 7:**
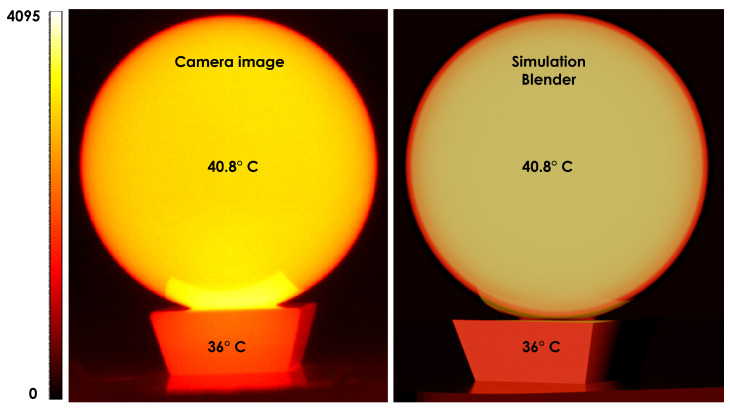
LWIR validation using a heated K9 glass sphere. The sphere and its glass support were heated in a climate chamber and imaged with a Ceres 640+ LWIR camera (Xenics, Leuven, Belgium). The scale uses arbitrary 12-bit values between 0 and 4095. operating over the 8–14 µm spectral range at measured temperatures of 40.8 °C and 36 °C, respectively. The synthetic LWIR rendering uses our Full Fresnel node with wavelength-dependent complex refractive indices and Planck-based emission, integrated over the LWIR band. The rendering reproduces the observed radiometric behaviour, including the temperature falloff toward the sphere’s edges due to the strong angular dependence of emissivity. Minor differences arise from the unknown spectral response and colour mapping of the thermal camera.

From the experimental dielectric graphs in [39], values for $\epsilon_{1}$ and $\epsilon_{2}$ were extracted by inter- and extrapolation for energies at both 20 meV (=62 micron) and 70 meV (=17.7 micron), like it was done in Axexandre et al. [40]. At 20 meV one obtains $\epsilon_{1}\approx$ −70, $\epsilon_{2}\approx$ 90 and at 70 meV we have $\epsilon_{1}\approx$ 60 and $\epsilon_{2}\approx$ 28. The corresponding (n,k)-pairs for these two sets of dielectric values are, by means of Equations (13), (4.69, 9.59) and (7.94, 1.76). The results of reflectances from air ($n_{i}=1$, $k_{i}=0$) to Bismuth, calculated with our approach are shown in Figure 5 and Figure 6). Because bismuth is strongly absorbing in the infrared, the imaginary part of the refractive index is non-negligible and directly affects the angular reflectance behavior. The agreement observed in Figure 5, Figure 6 and Figure 7 therefore critically depends on the use of complex refractive indices; a purely real-valued refractive index would not reproduce the measured reflectance trends. These plots closely resemble the plots in [40], which are overlayed in both Figure 5 and Figure 6, again validating the proposed Fresnel calculations in Section 2.3. The slight mismatch for the 20 meV reflectance data in Figure 5, can be attributed to the inaccuracy on the extrapolated dielectric values from the graph in [39].

The agreement in Figure 3, Figure 4, Figure 5 and Figure 6 is primarily assessed visually, as the reference curves are derived from experimentally measured dielectric data that are interpolated or extrapolated in wavelength and energy. Such reference data typically carry uncertainties on the order of a few percent, particularly in spectral regions with strong absorption or sparse sampling. The observed deviations between the proposed model and the reference results fall within this expected uncertainty range and do not indicate systematic errors in the Fresnel formulation.

### 5.3. Experimental Validation

To experimentally validate our model, we used LWIR simulations and experiments of an optical grade K9 crystal (spherical lens). The sphere was uniformly heated in a climate chamber for more than 3 h, to reach a starting temperature of 50 °C. Then camera images were taken with a Xenics Ceres 640+ LWIR camera operating from 8 to 14 µm, while the glass ball and its glass support were positioned out of the oven in an enclosed, almost non-reflecting environment. The moment we took the camera images, the central temperature of the glass ball and glass support were measured with a handheld thermometer indicating 40.8 °C for the ball and 36 °C for the support. The LWIR validation experiment was designed to qualitatively assess the angular radiometric behavior predicted by the proposed Fresnel-based emissivity model. Images were acquired using a Xenics Ceres 640+ LWIR camera operating in the 8–14 µm spectral band under standard factory settings. The camera was used in a relative radiometric mode; no absolute radiometric calibration, camera-specific spectral response, or proprietary color mapping was applied in the simulation.

The optical setup consisted of a stationary camera observing a spherical K9 glass element placed on a glass support at a fixed distance of approximately 0.5 m, with the optical axis aligned with the sphere center. The sphere was heated uniformly in a climate chamber for more than three hours to ensure thermal equilibrium prior to imaging. At the time of acquisition, the central temperatures of the sphere and support were measured using a handheld contact thermometer.

Figure 7 shows the result of putting these measured temperatures as parameters in our Full Fresnel material node for the modeled ball and support, while using wavelengths in the LWIR part of the spectrum. Although the validation in Figure 7 is presented as a thermal image rather than as explicit optical constants, the simulation relies on wavelength-dependent complex refractive indices of K9 glass. In particular, the imaginary part governs absorption and, via Kirchhoff’s law, the angular emissivity responsible for the observed radiometric falloff toward the sphere’s edges; using a purely real refractive index would not reproduce this behavior.

It should be noted that the comparison in Figure 7 does not correspond to a single wavelength or to an explicit wavelength–intensity spectrum. Both the measured and simulated images represent band-integrated LWIR radiance over the 8–14 µm range of the camera. In the simulation, wavelength-dependent Fresnel emissivity is evaluated across this spectral range and combined with Planck’s law, after which the radiance is integrated over wavelength. The exact spectral response function of the thermal camera was not available and is therefore not explicitly modeled. Again, we reached a remarkable resemblance between simulation and camera image, specifically correctly predicting the falloff of temperature towards the ball’s edges, because of a strong directional emissivity under large angles of incidence with respect to the local normals on the spherical surface. The major differences here are again attributed to the fact that we did not incorporate the full spectral response of the thermal camera and that we didn’t have knowledge on which typical main colors were used in the color scales of the camera.

## 6. Conclusions

The unified formulation presented in this work demonstrates that wavelength-dependent, complex-valued Fresnel behavior can be modeled robustly and consistently across the infrared spectrum. By directly incorporating measured complex refractive indices into a physically based rendering workflow, the method provides a general, material-agnostic interface model capable of handling both dielectrics and conductors within the same theoretical framework. This unified approach avoids the approximations commonly used in existing rendering systems and provides a stable foundation for accurate simulation of infrared light–matter interaction.

From a novelty perspective, the main contribution of this work is methodological rather than theoretical. By embedding a physically admissible and numerically stable Fresnel formulation into a general-purpose rendering engine, the work enables reproducible and physically grounded infrared simulations that were previously difficult to achieve with existing RGB- or approximation-based material models.

A key strength of the method lies in its robustness when applied to high-absorption (high-*k*) materials, where traditional Fresnel formulations often suffer from sign ambiguities, branch-cut inconsistencies, and numerical instabilities near grazing incidence. The proposed formulation overcomes these limitations by introducing a stable treatment of the complex square root and reflection coefficients, ensuring physically consistent predictions of reflectance and transmittance even in strongly absorbing infrared media.

From an application perspective, the model offers significant value to infrared system design. It enables precise evaluation of angle-dependent emissivity, reflection, and transmission in optical components, supporting tasks such as lens and window material selection, interface optimization, and radiometric calibration. For LWIR applications in particular—where absorption dominates and surface emissivity varies strongly with angle—the stability and physical correctness of the model are especially important. The ability to reproduce realistic directional emissivity effects, as shown in the experimental LWIR validation, underscores its relevance to thermal imaging, thermography, and long-wave system development.

The unified Fresnel model also provides notable advantages for multispectral imaging and synthetic data generation. By relying solely on complex refractive index data, it allows seamless transitions between spectral bands without changing the underlying shader model, making it well suited for constructing multispectral camera simulations, sensor-fusion pipelines, and spectrally parameterized scene renderings. Within 3D environments such as Blender, this capability bridges the gap between experimental optical measurements and synthetic image formation, enabling physically grounded virtual datasets for deep learning, defect detection, and algorithm benchmarking.

Despite its broad applicability, the approach remains subject to certain limitations. The current formulation assumes isotropic, homogeneous materials and does not include microfacet-based roughness, subsurface scattering, or polarization-dependent surface statistics. In addition, multilayer coatings, common in infrared optics, are not yet explicitly modeled. These simplifications restrict the accuracy of simulations involving rough surfaces, textured materials, or thin-film interference effects.

The validation materials selected in this work intentionally emphasize regimes in which absorption and complex-valued refractive indices play a decisive role, as these conditions are known to challenge conventional Fresnel implementations. Common optical polymers such as PMMA or polystyrene, which exhibit negligible extinction coefficients over much of the infrared spectrum, effectively reduce the Fresnel equations to their real-valued form and therefore do not stress the numerical or physical advantages of the proposed unified formulation. Anisotropic materials such as liquid crystals fall outside the isotropic-material assumptions of the present model and are left as future work.

Future work will focus on extending the unified Fresnel model to rough-surface BRDFs, integrating microfacet distributions compatible with complex-valued Fresnel coefficients, and supporting multi-layer coating stacks with wavelength-dependent phase propagation. These additions will further enhance the realism and predictive power of infrared simulations, expanding the range of scenarios that can be modeled within a physically based rendering framework.

## Data Availability

The data presented in this study are available on request from the corresponding author.

## Appendix A. Derivation of the Fresnel Reflectance and Transmittance

This appendix summarizes the derivation of the perpendicular and parallel Fresnel reflectances and transmittances used in the unified formulation presented in this work. The goal is to provide a compact but complete reference, consistent with the notation introduced in Section 3.2, and suitable for materials with wavelength-dependent complex refractive indices in the infrared.

### Appendix A.1. Snell–Descartes Law and Longitudinal Wave Vector

For an interface between two homogeneous, isotropic, non-magnetic media with complex refractive indices

$${\tilde{n}}_{i}=n_{i}+ik_{i},\;{\tilde{n}}_{t}=n_{t}+ik_{t},$$

the generalized Snell–Descartes law for complex media reduces to

(A1) $${\tilde{n}}_{i}\sin\theta_{i}={\tilde{n}}_{t}\sin\theta_{t}.$$

The longitudinal wave-vector component in the transmitted medium is defined as

(A2) $$A=\sqrt{{\tilde{n}}_{t}^{\;2}-{\tilde{n}}_{i}^{\;2}\sin^{2}\theta_{i}},$$

where the physically correct branch of the square root must be chosen such that

ℜ(A)≥0,ℑ(A)≥0,

ensuring attenuation rather than exponential growth inside an absorbing medium. This choice removes the sign ambiguity that often appears in naive evaluations of complex Fresnel formulas.

The auxiliary quantity in the incident medium is defined as

(A3) $$B={\tilde{n}}_{i}\cos\theta_{i}.$$

### Appendix A.2. Fresnel Reflection Coefficients

With the above definitions, the perpendicular and parallel Fresnel reflection coefficients take the compact forms

(A4) $$\begin{array}{cc} r_{⊥} & =\frac{B-A}{B+A}, \end{array}$$

(A5) $$\begin{array}{cc} r_{\|} & =\frac{{\tilde{n}}_{t}^{\;2}B-{\tilde{n}}_{i}^{\;2}A}{{\tilde{n}}_{t}^{\;2}B+{\tilde{n}}_{i}^{\;2}A}. \end{array}$$

These expressions are algebraically equivalent to the textbook Fresnel relations, but the use of *A* and *B* avoids repeated square-root operations and provides greater numerical stability, especially in materials with large extinction coefficients k(λ), as commonly found in LWIR applications.

### Appendix A.3. Reflectance and Transmittance

The energy reflectances are obtained as

(A6) $$\begin{array}{cc} R_{⊥} & =r_{⊥}r_{⊥}^{*}, \end{array}$$

(A7) $$\begin{array}{cc} R_{\|} & =r_{\|}r_{\|}^{*}, \end{array}$$

where ${\left(\cdot \right)}^{*}$ denotes the complex conjugate. The natural (unpolarized) reflectance is

(A8) $$R_{N}=\frac{1}{2}\left(R_{⊥}+R_{\|}\right).$$

The transmission coefficients can be written analogously as

(A9) $$\begin{array}{cc} t_{⊥} & =\frac{2B}{B+A}, \end{array}$$

(A10) $$\begin{array}{cc} t_{\|} & =\frac{2{\tilde{n}}_{i}{\tilde{n}}_{t}B}{{\tilde{n}}_{t}^{\;2}B+{\tilde{n}}_{i}^{\;2}A}. \end{array}$$

The corresponding energy transmittances must include the ratio of longitudinal wave-vector components:

(A11) $$\begin{array}{cc} T_{⊥} & =ℜ\;\left(\frac{A}{B}\right)\;t_{⊥}t_{⊥}^{*}, \end{array}$$

(A12) $$\begin{array}{cc} T_{\|} & =ℜ\;\left(\frac{A}{B}\right)\;t_{\|}t_{\|}^{*}. \end{array}$$

The factor ℜ(A/B) ensures continuity of the Poynting vector normal to the surface and is essential for complex-index media.

Using the auxiliary variables *A* and *B*, the Fresnel equations can be written in a compact, numerically stable form suitable for infrared modelling across SWIR–LWIR wavelengths. This formulation avoids ambiguities in the complex square root, preserves physical attenuation inside absorbing media, and ensures correct energy flow through the interface. These properties are essential for realistic infrared rendering, thermography simulations, and multispectral optical modelling in Blender’s node-based environment.
